# A Higher Liver Fibrosis-4 Index Is Associated With More Severe Hearing Loss in Idiopathic Sudden Sensorineural Hearing Loss

**DOI:** 10.7759/cureus.89864

**Published:** 2025-08-12

**Authors:** Yukihide Maeda, Soshi Takao, Ryotaro Omichi, Mizuo Ando

**Affiliations:** 1 Department of Otorhinolaryngology, Saitama Medical University, Saitama, JPN; 2 Department of Otolaryngology-Head and Neck Surgery, Okayama University Graduate School of Medicine, Dentistry and Pharmaceutical Sciences, Okayama, JPN; 3 Department of Epidemiology, Okayama University Graduate School of Medicine, Dentistry and Pharmaceutical Sciences, Okayama, JPN

**Keywords:** aspartate aminotransferase-to-platelet ratio index, audiometry, fatty liver disease, incidence, liver fibrosis-4 index, severity, sudden hearing loss

## Abstract

Background

Liver fibrosis is an important medical issue increasing over time in developed countries.

Aims/objectives

This study aimed to investigate whether liver fibrosis, as indicated by routine blood test parameters, influences the risk and severity of idiopathic sudden sensorineural hearing loss (ISSNHL).

Material and methods

Sixty-six patients with ISSNHL and 198 patients with benign parotid gland tumors (BPTs) (controls) were enrolled. Indices for liver fibrosis (Liver Fibrosis-4 index (FIB-4 index) and aspartate aminotransferase-to-platelet ratio index (APRI)) were calculated from the blood laboratory data. The pure tone average (PTA) was calculated as the mean of hearing levels at the six frequencies at the onset of ISSNHL. Severe hearing loss was defined as PTA≥60 decibels Hearing Level (dB HL).

Results

In risk evaluation, the FIB-4 index did not differ significantly between ISSNHL patients and controls. Regarding the severity of ISSNHL, the FIB-4 index was significantly higher in ISSNHL patients with severe hearing loss than in those with PTA<60 dB HL (P<0.05) on univariate comparison. After adjusting for age, sex, and indices of inflammation, both the FIB-4 index and APRI showed a significant association with severe hearing loss (odds ratio (OR): 5.9, 95% confidence interval (CI): 1.3-25.7, and OR: 2.2, 95% CI: 1.1-4.7).

Conclusions and significance

Higher liver fibrosis indices (FIB-4 index and APRI), derived from routine blood laboratory data, are associated with a more severe phenotype of ISSNHL.

## Introduction

Idiopathic sudden sensorineural hearing loss (ISSNHL) is generally considered a multifactorial disease. The pathological mechanisms of ISSNHL are largely unknown, partly because of a technical obstacle: it is difficult to biopsy the inner ear tissues of the patients. ISSNHL does not lead to mortality, and the postmortem temporal bone pathology can be examined only a considerable time after the onset of ISSNHL [1].

Liver fibrosis associated with non-alcoholic fatty liver disease (NAFLD) is an important medical issue increasing over time in developed countries. The prevalence of advanced liver fibrosis due to NAFLD increased from 0.84% to 1.75% from 1999-2002 to 2009-2012 in the United States [2]. The Liver Fibrosis-4 index (FIB-4 index) is a non-invasive prediction score for fatty liver diseases (NAFLD and non-alcoholic steatohepatitis) calculated from blood laboratory tests [3]. The FIB-4 index is calculated from the patients’ age and laboratory data of aspartate transaminase (AST), alanine transaminase (ALT), and platelets (PLT), and predicts liver fibrosis/cirrhosis confirmed by liver biopsy with an area under the curve (AUC) of 0.81, sensitivity of 0.57, and specificity of 0.89. The aspartate aminotransferase-to-platelet ratio index (APRI) is also a blood-based biomarker employed to predict significant liver fibrosis and cirrhosis, as verified by liver biopsy [3].

In general, cardiovascular risk factors such as abnormal body mass index, diabetes mellitus, hypertension, total cholesterol, low-density lipoprotein cholesterol, and a medical history of myocardial infarction are associated with the risk of ISSNHL [4,5]. Early signs of chronic liver disease on magnetic resonance imaging (MRI) were associated with increased cardiovascular disease risk [6]. Liver fibrosis assessed by the FIB-4 index was associated with atrial fibrillation in acute ischemic stroke patients [7].

To date, there have been few papers showing associations between fatty liver diseases and ISSNHL [8,9]. The present study examined how the FIB-4 index score is associated with the risk and severity of ISSNHL, to gain insights into how liver fibrosis is involved in the development of ISSNHL.

## Materials and methods

This was a retrospective, observational study. The protocol was approved by the Ethics Review Board of Okayama University Hospital (protocol number: 2010-026) and performed in compliance with the Declaration of Helsinki. Informed consent was regarded to have been acquired by providing information on the ability to opt out on the homepage of the Otolaryngology Department, Okayama University Hospital.

Study participants

The medical records of inpatients at the Department of Otolaryngology at Okayama University Hospital between October 2003 and May 2022 were retrospectively reviewed. The inclusion criteria for ISSNHL patients were as follows: both female and male patients of all ages who developed sudden sensorineural hearing loss of unknown cause and blood laboratory tests performed before corticosteroid treatment. Patients with middle ear diseases and/or conductive hearing loss were excluded. The diagnosis of ISSNHL followed the 2012 criteria of the American Academy of Otolaryngology-Head and Neck Surgery, except that the patients showing hearing loss < 30 dB were also included in the analyses because the present study focused on the severity of hearing loss, and the hearing loss of the patients ranged from mild to severe [10]. Most patients included in this study were treated with intravenous corticosteroid therapy, which was initiated within two weeks after the onset of ISSNHL. The laboratory data of inpatients with benign parotid gland tumors (BPTs) were collected as the control. These patients underwent surgical resection, and the blood laboratory tests were performed to assess the indication for surgical resection of the BPTs. To date, evidence supporting any association between BPTs and metabolic disorders remains scarce. An epidemiological study conducted in South Korea found no significant association between BPTs and obesity [11].

Collection of laboratory data

**Figure 1:**
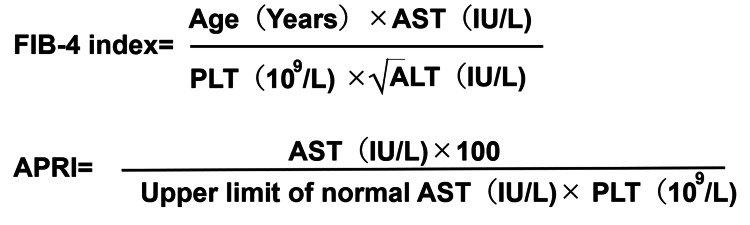
Formulae of the FIB-4 index (top) and APRI (bottom) FIB-4 index: Liver Fibrosis-4 index, APRI: aspartate aminotransferase-to-platelet ratio index, AST: aspartate transaminase, ALT: alanine transaminase, PLT: platelet

Blood laboratory tests were performed at the time of hospitalization and before starting any corticosteroid therapy. The data for white blood cell (WBC) count, neutrophil count, lymphocyte count, AST, ALT, and PLT were collected from the medical charts. In general, the inflammatory reaction in the cochlea is involved in the pathology of ISSNHL, and it was reported that the neutrophil-to-lymphocyte ratio (NLR) was higher in ISSNHL patients at disease onset than in controls [12]. For this reason, WBC count and NLR were included as the adjusted variables that may be associated with the risk and severity of ISSNHL. The FIB-4 index was calculated using patient age and laboratory data (AST, ALT, and PLT), according to the formula presented in Figure 1 (upper formula). The APRI was calculated using laboratory data (AST, ALT, and PLT), according to the formula shown in Figure 1 (lower formula).

Collection of audiometric data

Audiometric data were obtained at the onset of ISSNHL and before starting any corticosteroid therapy. The pure tone average (PTA in decibels Hearing Level (dB HL)) was calculated as the mean value of the six air-conduction thresholds at 250, 500, 1,000, 2,000, 4,000, and 8,000 Hz. The patients were divided into two groups showing PTA<60 dB HL and PTA≥60 dB HL. Severe hearing loss was defined as PTA≥60 dB HL. In this study, participants with both severe and non-severe hearing loss were included. Consequently, some individuals presented with hearing loss characterized by either an ascending or a descending audiogram. To ensure comprehensive coverage across the frequency spectrum, the average hearing threshold was calculated as the mean value of six frequencies. According to the severity classification for ISSNHL established by the Research Committee of the Ministry of Health, Labour and Welfare of Japan, grade 3 hearing loss is defined as a threshold of ≥60 dB and <90 dB [13]. Accordingly, in the present study, a hearing threshold of ≥60 dB was adopted as the criterion for severe hearing loss.

Statistical analysis

First, the FIB-4 index was compared between ISSNHL patients and control patients (Student’s t-test). Next, the FIB-4 index was compared between ISSNHL patients with PTA<60 dB HL and PTA≥60 dB HL (Student’s t-test).

Logistic regression models were employed to estimate the adjusted odds ratios (ORs) and 95% confidence intervals (CIs) for the explanatory variables (FIB-4 index (high versus low, as defined below) and APRI (per 0.1-unit increment)) in relation to the dependent variable of severe hearing loss. Adjustments were made for sex (female; reference: male), age, WBC count (per 1,000/μL increment), and neutrophil-to-lymphocyte ratio (NLR; neutrophil count divided by lymphocyte count). In general, older ISSNHL patients are more predisposed to severe hearing loss than younger patients [14]. However, patients’ age was not directly included as an adjusted variable because the formula for the FIB-4 index includes patient age, and these two variables may cause multicollinearity in the regression model. As an independent variable, the FIB-4 index was dichotomized into low (FIB-4 index<1.3, reference) and high (FIB-4 index≥1.3), because an FIB-4 cutoff value of 1.3 can differentiate the prognosis of liver diseases, such as all-cause mortality, hepatocellular carcinoma, liver transplantation, or cirrhosis [15]. Statistical analyses were performed using SPSS version 28 software (IBM Corp., Armonk, NY).

## Results

Sixty-six ISSNHL patients (29 female and 37 male patients, 52.6±19.7 (mean±SD) years old) and 198 controls (102 female and 96 male patients, 56.7±15.5 years old) were identified during the study period. There was no significant difference in the male-to-female ratio between patients with ISSNHL and the control group (P>0.05 by chi-squared test). Patient age was not significantly different between the ISSNHL patients and controls (P>0.05 by t-test). The mean PTA in the affected ear at the onset of ISSNHL was 68.7±27.1 dB HL. Twenty-two ISSNHL patients with PTA<60 dB HL (mean PTA: 36.8±11.9) and 44 ISSNHL patients with PTA≥60 dB HL (mean PTA: 84.7±16.2) were identified. Patients with PTA<60 dB HL (45.9±19.3 years old) were significantly younger than those with PTA≥60 dB HL (56.1±19.2 years old) (P<0.05 by t-test).

**Figure 2:**
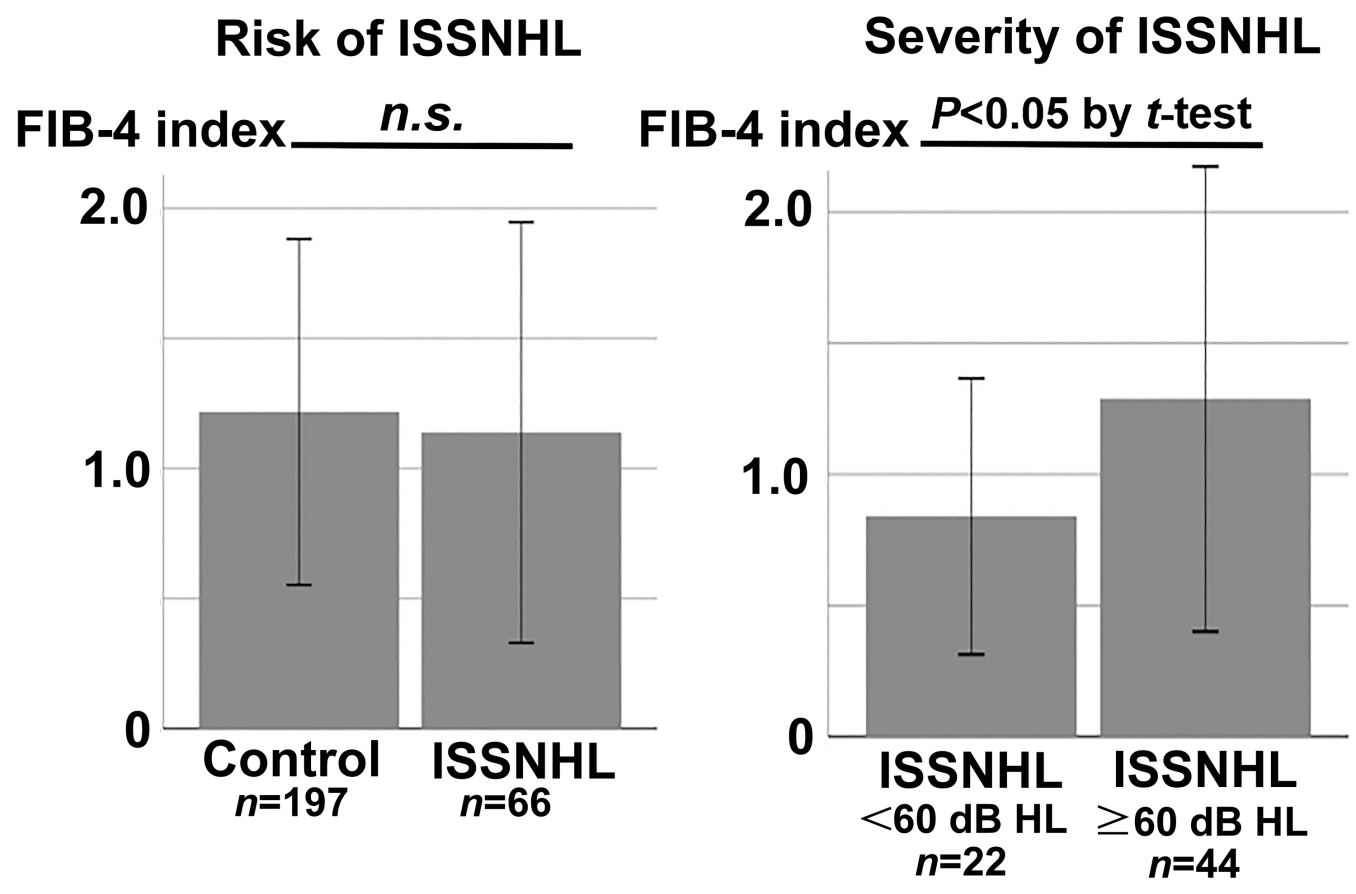
FIB-4 indices compared between the ISSNHL and control groups (left), and between patients with PTA<60 dB HL and PTA≥60 dB HL (right) Bars represent the mean±SD. FIB-4: Liver Fibrosis-4, ISSNHL: idiopathic sudden sensorineural hearing loss, PTA: pure tone average, SD: standard deviation

The FIB-4 index was not significantly different between the ISSNHL patients (1.14±0.81, n=66) and controls (1.22±0.66, n=197) (P>0.05) (Figure 2, left). The FIB-4 index was significantly higher in ISSNHL patients with PTA≥60 dB HL (1.29±0.89, n=44) than in those with PTA<60 dB HL (0.84±0.53, n=22) (P<0.05) (Figure 2, right).

Table 1 and Table 2 show the results of the logistic regression models. After taking into account potential confounding variables, both the FIB-4 index (OR: 5.9, 95% CI: 1.3-25.7) and the APRI (OR: 2.2, 95%CI: 1.1-4.7) showed significant associations with severe hearing loss. Of the adjusted variables, lower NLR showed significant associations with severe hearing loss in the model, including the FIB-4 index. Lower NLR and higher WBC count showed significant associations with severe hearing loss in the model, including the APRI index.

**Table 1:** Logistic regression model with the outcome of severe hearing loss (PTA≥60 dB HL) and independent variable of FIB-4 index Using logistic regression modeling (chi-squared test for the model, P<0.05), the FIB-4 index was positively associated with a higher risk of severe hearing loss. The indices of inflammation (white blood cells and the neutrophil-to-lymphocyte ratio) also showed significant or marginally significant associations with severe hearing loss. PTA: pure tone average, FIB-4 index: Liver Fibrosis-4 index, CI: confidence interval, WBC: white blood cell

Variables	Odds ratio (95% CI)	P value
FIB-4 index (cutoff value: 1.3)	5.9 (1.3-25.7)	0.02
Neutrophil-to-lymphocyte ratio	0.7 (0.6-0.98)	0.04
WBC	1.4 (0.98-2.1)	0.06
Sex	1.6 (0.5-5.0)	0.45

**Table 2:** Logistic regression model with the outcome of severe hearing loss (PTA≥60 dB HL) and independent variable of the APRI Using logistic regression modeling (chi-squared test for the model, P<0.01), the APRI was positively associated with a higher risk of severe hearing loss. The indices of inflammation (white blood cells and the neutrophil-to-lymphocyte ratio) also showed significant associations with severe hearing loss. PTA: pure tone average, APRI: aspartate aminotransferase-to-platelet ratio index, CI: confidence interval, WBC: white blood cell

Variables	Odds ratio (95% CI)	P value
APRI (per 0.1 increment)	2.2 (1.1-4.7)	0.04
Neutrophil-to-lymphocyte ratio	0.7 (0.5-0.9)	0.02
WBC	1.8 (1.1-3.0)	0.02
Age	1.04 (1.002-1.07)	0.04
Sex	2.3 (0.6-8.5)	0.2

## Discussion

In the present study, the FIB-4 index did not differ between ISSNHL patients and controls; therefore, there was no evidence suggesting that liver fibrosis was associated with the risk of ISSNHL. In general, atherosclerosis and cardiovascular risk factors are associated with the risk of ISSNHL [5], and the most likely causes of ISSNHL are vascular impairments such as cochlear ischemia, cochlear infarction, and cochlear haemorrhage [16]. The underlying risk factors for liver fibrosis, such as dyslipidemia and glucose metabolism dysregulation, also contribute to the pathogenesis of atherosclerotic cardiovascular disease [17]. However, there was no association between a higher FIB-4 index and the risk of ISSNHL. Based on these data, liver fibrosis may not be the cause of ISSNHL.

The present univariate and multivariate analyses showed that a higher FIB-4 index was associated with more severe hearing loss in ISSNHL patients. An FIB-4 index of ≥1.3 suggests the presence of liver fibrosis and is associated with more severe hearing loss in ISSNHL patients (≥60 dB). In a previous study by Tatoli et al., the more severe phenotype of age-related hearing loss (as the independent variable) was associated with a higher risk of liver fibrosis (FIB-4 index of ≥2.67 as the dependent variable) in community residents (total: 1,929 persons) in Italy [18]. Although the regression model in the study of Tatoli et al. differed from the present logistic regression model, both studies showed a significant relationship between a higher FIB-4 index and the more severe phenotype of sensorineural hearing loss. While the study by Tatoli et al. demonstrated an association between the severity of age-related hearing loss and elevated FIB-4 index values, the present study demonstrated a relationship between the severity of ISSNHL and elevated FIB-4 index values. Liver fibrosis is a consequence of profound systemic dysregulation in lipid metabolism with risk factors such as diabetes mellitus, hypertriglyceridemia, and excess visceral adiposity. It was previously shown that diabetes mellitus and high triglyceride levels were associated with the more severe phenotype of ISSNHL [14]. Therefore, exacerbations of these risk factors for liver fibrosis also lead to the more severe phenotype of ISSNHL. However, it remains to be determined how liver fibrosis affects the pathological mechanisms of ISSNHL and causes the severe phenotype of hearing loss as a consequence of liver dysfunction.

A recent (2025) epidemiological study by Kang et al. reported that older adults with metabolic dysfunction-associated steatotic liver disease may have an increased risk of developing ISSNHL [9]. Kang et al. proposed potential mechanisms linking liver disease to ISSNHL, including the induction of systemic inflammation due to hepatic injury, which in turn triggers the production of inflammatory cytokines [19], and systemic oxidative stress impairing mitochondrial function, thereby damaging the auditory nerve and inner ear [20].

In general, older age has a significant impact on the risk and severity of ISSNHL. Therefore, it was necessary to exclude the possibility that the association between a higher FIB-4 index and the more severe phenotype of ISSNHL merely reflected the contribution of aging in the pathogenesis of ISSNHL. To this end, the relationship between the severity of ISSNHL and APRI, an index that does not include the patient’s age in its formula, was examined. It was demonstrated that a higher APRI was associated with a more severe phenotype of ISSNHL, showing that liver fibrosis was clearly related to the more severe phenotype of ISSNHL.

Limitations of the study

The retrospective, single-center design and small sample size, particularly in the severe hearing loss group, may limit statistical power and generalizability. Future large-scale prospective studies in patients with liver fibrosis may help elucidate the potential association between liver fibrosis and ISSNHL. Ideally, the association between liver fibrosis and ISSNHL should be demonstrated using data from a large-scale prospective study, with adjustments made for comorbidities such as diabetes, hypertension, and hyperlipidemia. Future studies should include data from patients with liver fibrosis confirmed by biopsy or other definitive diagnostic methods. This is important because the FIB-4 index and APRI may not be specific to the liver and can be elevated as a result of systemic inflammation or infection, which may also contribute to the pathophysiology of ISSNHL. The control group of BPT patients may not reflect the general population’s liver fibrosis distribution, potentially affecting risk estimates. As a cross-sectional study, temporality and causality cannot be established; higher FIB-4 index scores may be a marker rather than a cause of more severe hearing loss. Laboratory measurements for FIB-4 index and APRI were taken at a single time point and may have been influenced by acute illness or treatment. Finally, although we speculate on vascular and metabolic mechanisms linking liver fibrosis and cochlear dysfunction, no direct mechanistic measures were included.

## Conclusions

Liver fibrosis has become an increasingly prevalent health concern in developed countries. This study aimed to investigate whether the presence of liver fibrosis influences the risk and clinical severity of ISSNHL. In the assessment of risk, no significant difference in the FIB-4 index was observed between patients with ISSNHL and control subjects. However, in evaluating the severity of ISSNHL, univariate analysis showed that patients with more profound hearing loss exhibited significantly higher FIB-4 index values compared to those with a pure tone average below 60 dB HL. Multivariate analysis, adjusted for age, sex, and inflammatory markers, revealed that both the FIB-4 index and APRI were significantly associated with greater hearing loss severity. Liver fibrosis indices may serve as potential markers for stratifying disease severity at presentation, although their prognostic utility requires confirmation in larger, prospective studies. Further investigation into the pathophysiological mechanisms underlying this association is warranted.
